# Caveolin-1 in Lipid Rafts Interacts with Dengue Virus NS3 during Polyprotein Processing and Replication in HMEC-1 Cells

**DOI:** 10.1371/journal.pone.0090704

**Published:** 2014-03-18

**Authors:** Julio García Cordero, Moisés León Juárez, Jorge A. González-Y-Merchand, Leticia Cedillo Barrón, Benito Gutiérrez Castañeda

**Affiliations:** 1 Departamento de Biomedicina Molecular, Centro de Investigación y Estudios Avanzados del IPN, México City, México; 2 Departamento de Microbiología, Escuela Nacional de Ciencias Biológicas IPN, México City, México; 3 Laboratorio de Inmunología, Facultad de Estudios Superiores Iztacala Universidad Autónoma de México, Tlalnepantla Estado de México, México; SRI International, United States of America

## Abstract

Lipid rafts are ordered microdomains within cellular membranes that are rich in cholesterol and sphingolipids. Caveolin (Cav-1) and flotillin (Flt-1) are markers of lipid rafts, which serve as an organizing center for biological phenomena and cellular signaling. Lipid rafts involvement in dengue virus (DENV) processing, replication, and assembly remains poorly characterized. Here, we investigated the role of lipid rafts after DENV endocytosis in human microvascular endothelial cells (HMEC-1). The non-structural viral proteins NS3 and NS2B, but not NS5, were associated with detergent-resistant membranes. In sucrose gradients, both NS3 and NS2B proteins appeared in Cav-1 and Flt-1 rich fractions. Additionally, double immunofluorescence staining of DENV-infected HMEC-1 cells showed that NS3 and NS2B, but not NS5, colocalized with Cav-1 and Flt-1. Furthermore, in HMEC-1cells transfected with NS3 protease, shown a strong overlap between NS3 and Cav-1, similar to that in DENV-infected cells. In contrast, double-stranded viral RNA (dsRNA) overlapped weakly with Cav-1 and Flt-1. Given these results, we investigated whether Cav-1 directly interacted with NS3. Cav-1 and NS3 co-immunoprecipitated, indicating that they resided within the same complex. Furthermore, when cellular cholesterol was depleted by methyl-beta cyclodextrin treatment after DENV entrance, lipid rafts were disrupted, NS3 protein level was reduced, besides Cav-1 and NS3 were displaced to fractions 9 and 10 in sucrose gradient analysis, and we observed a dramatically reduction of DENV particles release. These data demonstrate the essential role of caveolar cholesterol-rich lipid raft microdomains in DENV polyprotein processing and replication during the late stages of the DENV life cycle.

## Introduction

Dengue viruses (DENVs) are enveloped, positive-sense RNA viruses that belong to the Flaviviridae family.DENVs initiate their life cycle through receptor-mediated endocytosis at the cellular membrane. After internalization, the conformation of the viral envelope protein changes to promote the release of the genome into the cytoplasm. The genome is translated into a large polyprotein that is proteolytically processed to yield three structural proteins (envelope protein, membrane precursor protein, and capsid) and seven non-structural (NS) proteins (NS1, NS2A, NS2B, NS3, NS4A, NS4B, and NS5) [1], [2].

During processing, the polyprotein is cleaved by NS3 protease and host proteases in the lumen of the endoplasmic reticulum (ER). NS3 requires NS2B as a cofactor to produce mature proteins. NS3, along with NS5, is also involved in the DENV replication complex. NS3 possess RNA helicase and nucleotide triphosphatase activities. NS5 contains a methyltransferase domain and an RNA polymerase domain [3]–[5].Dengue virus induces the remodeling and redistribution of distinct membrane structures to obtain a platform for viral RNA replication, assembly, and spreading, [6], [7]. Recent research using electron tomography techniques has demonstrated that viral replication occurs on double-membrane vesicles adjacent to the ER. Furthermore, image analyses have shown physical linkages between the sites of DENV replication and assembly [8].

Cellular membranes contain organized assemblies of different lipids (glycerophospholipids, sphingolipids, and cholesterol) and different proteins that cluster together in the cell membrane within discrete microdomains known as lipid rafts. In normal cells, lipid rafts participate in the re-arrangement and trafficking of membrane-associated proteins and promote cell signal transduction by recruiting necessary molecules [9], [10].

The localization of viral structural proteins and the effects of raft-disrupting agents on the replication of several viruses, including DENV, hepatitis C virus [11], and West Nile virus, have demonstrated the involvement of lipid rafts in viral entry [12], [13]. During viral entry, lipid rafts may serve as platforms that recruit viral receptors and then transport the virus to the appropriate intracellular compartment [14], [15]. Previous research has shown that flaviviral entry, RNA uncoating, and replication are blocked by the removal or addition of cholesterol, which suggests that minor changes in the cholesterol concentration of target membranes are required for productive flaviviral infection [16]. However, the role of lipid raft membranes in the protein processing, replication, or assembly of DENV has not been well characterized.

There are two types of lipid rafts, caveolar and non-caveolar, which contain caveolin (Cav-1) or reggie proteins (flotillins), respectively. Non-caveolar rafts contribute to clathrin-independent endocytosis, and both types of rafts are involved in protein trafficking, cholesterol homeostasis, and signaling [17], [18]. Ectopic expression of Cav-1 specifically suppresses the replication of human immunodeficiency virus (HIV)-1 [19]. Evidence from a recent study strongly suggests that Cav-1 inhibits HIV-1 transcription through a nuclear factor-kappaB (NF-κB)-dependent mechanism [20]. HIV-1 infection induces a number of signal transduction pathways, some of which involve Cav-1. Therefore, Cav-1 likely creates a restrictive cellular environment for HIV-1 replication [21]–[23].

In this study, we investigated the significance of caveolar and non-caveolar lipid rafts during the late stages of the DENV life cycle, including polyprotein processing and replication of DENV-2. Membrane flotation and co-immunoprecipitation analyses indicated that a fraction of NS3 likely associates with Cav-1 during polyprotein processing. Fluorescence microscopy analysis of virus-infected human microvascular epithelial cells (HMEC-1) showed that NS3 and NS2B, but not NS5, colocalize with Cav-1 and flotillin 1 (Flt-1) present in lipid raft membranes. Furthermore, the NS3 protease domain and NS2BNS3pro, but not NS2B, overlapped with Cav-1 protein when over expressed in HMEC-1 cells. In contrast, viral double-stranded RNA (dsRNA) overlapped weakly with Cav-1 and flotillin. The data show that Cav-1 plays a role in the late stages of the viral life cycle.

## Results

### Characterization and expression of major lipid raft markers in HMEC-1 cells

**Figure 1:**
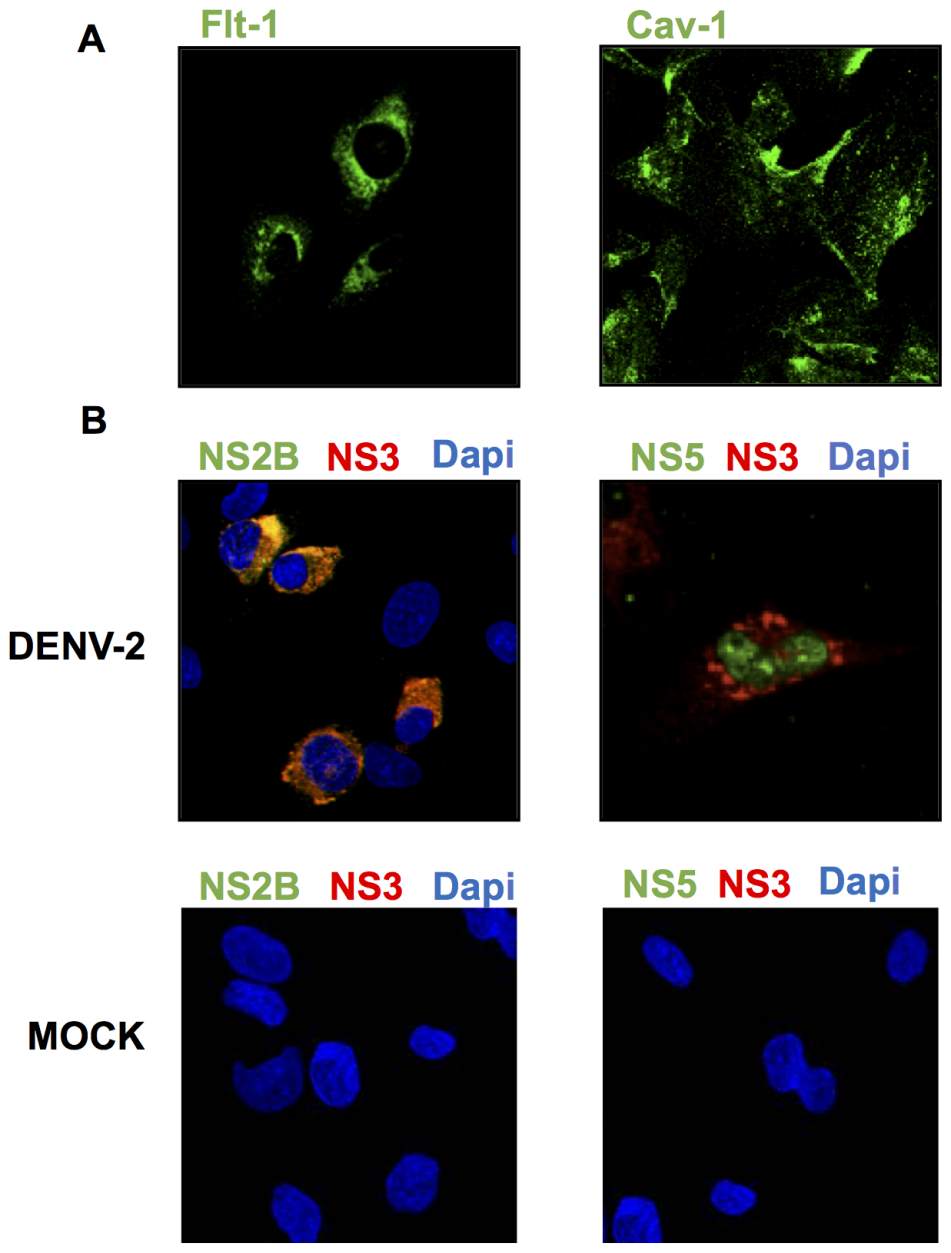
Localization of lipid raft resident proteins and viral DENV proteins. (A) HMEC-1cells were stained with specific antibodies against Cav-1 and Flt-1, and immunofluorescence were analyzed by confocal laser scanning microscopy. (B) HMEC-1 cells were mock infected or infected with DENV-2 and then double-stained with specific antibodies (developed in our laboratory) targeting NS2B (green) and NS3 (red) or NS3 (red) and NS5 (green).

To confirm the cellular distribution of the lipid raft protein markers Cav-1 and Flt-1 in our model, HMEC-1cells were stained with anti-Cav-1 and anti-Flt-1 antibodies. The distribution of Flt-1 was perinuclear; whereas Cav-1 was broadly distributed throughout the cytoplasm (Fig. 1A). To assert the subcellular localization of the DENV non-structural proteins NS2B, NS3, and NS5, which are involved in polyprotein processing and viral RNA replication HMEC-1 cells were infected with 10 MOI DENV and analyzed 48 hours post infection. As expected, NS3 and NS2B localization was perinuclear in small clusters. In contrast, a very low level of NS5 was observed in the cytoplasm; most NS5 was concentrated in the nucleus 48 h postinfection. The infected cells were also double stained for the viral proteins NS3 and NS2B. As presumed, NS3 and NS2B colocalized in the perinuclear region (Fig. 1B). This colocalization was predicted considering that the NS2B is a cofactor of NS3. However, although NS3 and NS5 are part of the replicative complex, not all NS3 overlapped with NS2B, and none overlapped with NS5. Therefore, it is important to consider the different functions of the NS3 protein, such as polyprotein processing, as well as its role in RNA replication. No signals were observed in mock-infected cells.

### NS3 and NS2B, but not NS5, is present in detergent-resistant microdomains (DRMs) in HMEC-1cells

**Figure 2:**
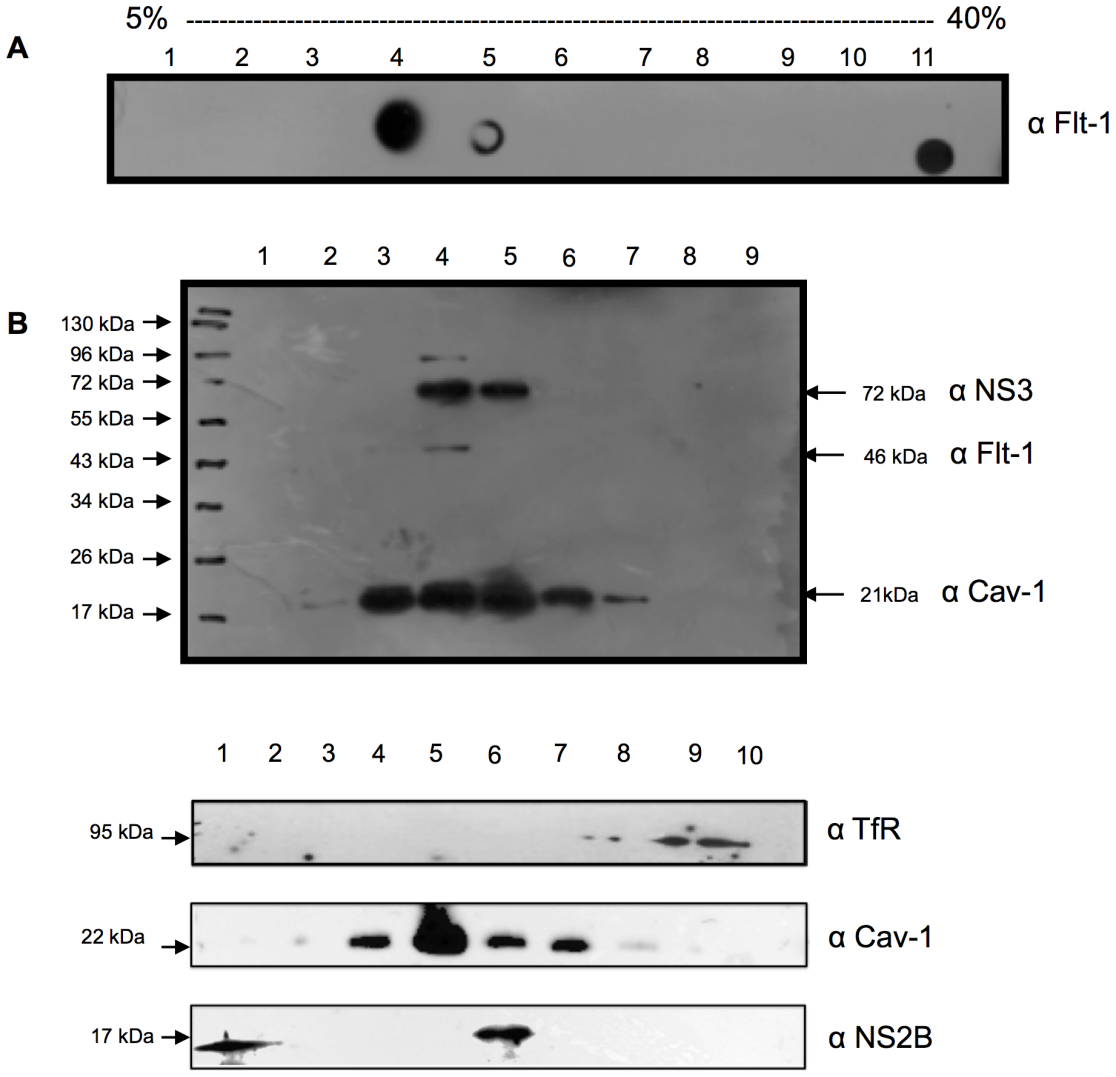
NS3 and NS2B are present in detergent-resistant membrane fractions. HMEC-1cells were infected with DENV-2 at 10 MOI for 48 h and lysed using 1% Brij in TNEV buffer. Lysates were analyzed by sucrose gradient ultracentrifugation (A). The floating bands from sucrose gradient ultracentrifugation were collected and analyzed by dot blot with an anti-Flt-1 antibody (a lipid resident protein). (B) The samples from each fraction were then analyzed by western blotting using specific antibodies against NS3, NS2B, Flt-1, Cav-1 and TfR as a marker of non-raft fractions.

DENV polyprotein processing, replication, and assembly occur in intracellular membrane structures [16]. Based on the specific properties of DRMs, non-caveolar or caveolar rafts appear to be suitable structures for supporting different steps of the viral cycle. To address whether the DENV proteins NS2B, NS3, and NS5 localized in DRMs, HMEC-1cellswere infected with DENV, and membrane and cytosolic fractions were separated by sucrose gradient ultracentrifugation each fraction was analyzed by dot blot with a marker protein of lipid rafts anti-Flt1 (Fig 2A). To confirm that the floating opaque band observed at the interface between 5% and 30% sucrose contained lipid raft microdomains, equal volumes of each fraction were analyzed by western blotting using the following markers: Cav-1, Flt-1, and transferrin receptor (TfR). As shown in Fig. 2B, NS2B and NS3 appeared in the same fraction as Cav-1 and Flt-1. This fraction did not contain TfR, a non-raft membrane protein, which was observed in fractions 10 and 11. Thus, NS3 partitioned to fractions containing DMRs. Although markers for both types of rafts were identified in this zone, the proportion of caveolar (Cav-1-positive) rafts was greater than the proportion of non-caveolar (Flt-1-positive) rafts, confirming that HMEC-1 cells were enriched with caveolar rafts.

### NS3 and NS2B, but not NS5, colocalize with major lipid raft proteins on cellular membranes

**Figure 3:**
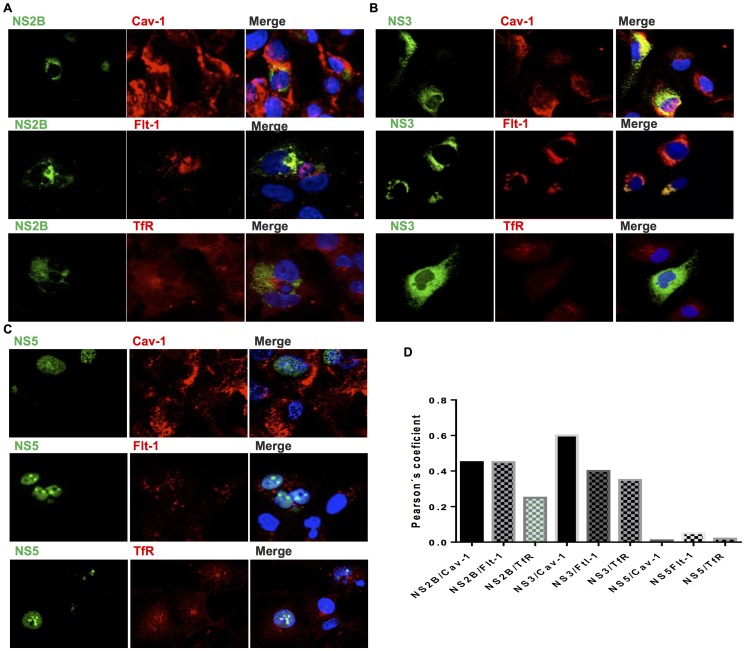
Colocalizationanalysis of DENV NS2B, NS3, and NS5 with caveolin and flotillin in infected cells. HMEC-1 cells were mock infected or infected with DENV-2, fixed 36 h after infection with 4% paraformaldehyde, permeabilized, and immunolabeled. Cells were double stained with mouse monoclonal antibodies against NS2B (A), NS3 (B), or NS5 (C); the lipid raft markers Cav-1 (upper panel) and Flt-1 (middle panel); and the non-raft marker TfR (bottom panel). DAPI was used for nuclear DNA staining. Slides were analyzed by confocal laser scanning microscopy. (D) The degree of overlap between the green and red signals for each viral protein was statistically analyzed and expressed as a Pearson's coefficient by the microscopy software.

Little is known about the role of lipid rafts as platforms during DENV polyprotein processing or replication [16]. Thus, HMEC-1 cells were mock infected or infected with DENV-2 at 10 MOI, and colocalization analysis was performed for the viral proteins NS2B, NS3, NS5, Cav-1, Flt-1, and TfR. At 36 h post infection, there was a clear colocalization of the NS2B protein with Cav-1 and Flt-1 in a high percentage of cells (Fig. 3A), although the distribution varied slightly between cells. NS3 also colocalized with Cav-1 and Flt-1, but to a greater extent than NS2B (Fig. 3B). Most overlapping signal was observed as a punctuate pattern in a structure that resembled the ER; the rest of the signal was observed in different cellular sites. In contrast, NS5 polymerase was observed mainly in the nucleus, with a weak signal in the cytoplasm (Fig.3C). Interestingly, no clear overlap was observed with Cav-1 and Flt-1. TfR did not colocalize with NS3, NS2B, or NS5 (Fig. 3A–C, bottom). Similar patterns were found in at least five different fields in four independent experiments.

**Figure 4:**
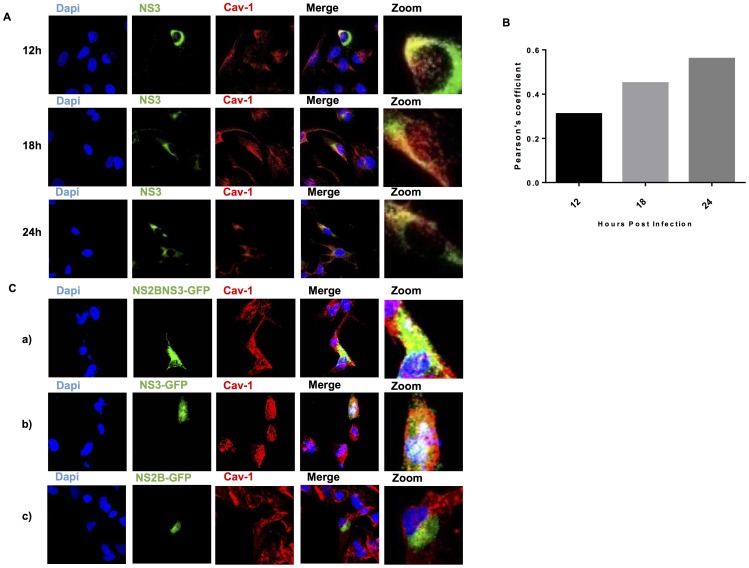
DENV-2 NS3 with Cav-1 colocalize at early stages after infection. (A) HMEC-1cells were infected with DENV-2 and double stained at different times after infection (12, 24 and 48hpi) or were mock infected (not shown). In all cases, cells were double stained with mouse monoclonal antibodies against NS3 (green) and the lipid raft marker Cav-1 (red). Nuclear DNA was stained with DAPI. (B) Slides were analyzed by confocal laser scanning microscopy (Leica SP5 Objective HCXPLAPO63 63Oil). The graph shows the colocalization index. (C) HMEC-1 cells were transfected with (a) NS2BNS3pro-GFP, (b) NS3pro-GFP, and,(c) NS2B-GFP. After 24 h, they were stained with the lipid raft marker Cav-1 (red), and colocalization was analyzed.

Because the strongest colocalization was observed between Cav-1 and NS3 by statistical Pearson analysis (Fig 3D), we assessed whether the colocalization of NS3 and Cav-1 was a transient phenomenon that occurred late in infection. Cav-1 and NS3 colocalization appeared as early as 12 h post infection and increased until 24 h post infection (Fig. 4A). In the graphical results, the Pearson index for the NS3 protein is higher than for the rest of the proteins (Fig. 4B). Taken together, these results suggest that NS3 and, to a lesser extent, NS2B associate with lipid rafts during DENV processing and RNA replication in infected cells. The consistent association of NS3 with Cav-1 at different times post infection suggests the involvement of lipid rafts in the late steps of infection.

### NS2B, NS3pro, and the NS2BNS3pro complex colocalize with Cav-1 lipid rafts

To determine whether polyprotein processing occurred in caveolar lipid rafts and if the protease functional domain was responsible for lipid raft colocalization, we transfected HMEC-1 cells with plasmids encoding NS3pro-green fluorescent protein (GFP), NS2BNS3pro-GFP, or NS2B-GFP and stained the cells with anti-Cav-1 antibodies 24 h later. Cav-1 colocalized strongly with NS3pro-GFP and the NS2BNS3-GFP complex, but weakly colocalized with NS2B-GFP. These results indicate that the NS3 protease region, when transiently transfected into HMEC-1 cells, localizes to lipid rafts through colocalization with Cav-1 in the absence of NS2B (Fig. 4C a–c).

### dsRNA weakly colocalizes with lipid rafts

**Figure 5:**
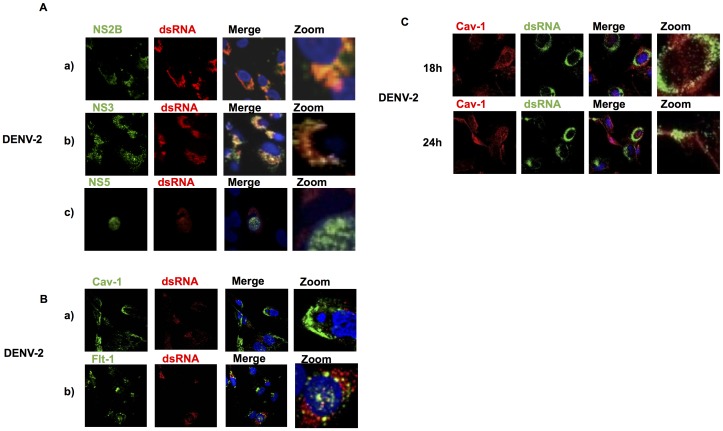
dsRNA weakly colocalizes with lipid raft markers in DENV-infected HMEC-1. (A) HMEC-1 cells, mock infected or infected with DENV-2, were fixed 48 h after infection, permeabilized, and analyzed by immunofluorescence. Cells were double stained with mouse monoclonal antibodies against dsRNA (red always) and NS2B (green) (a); dsRNA and NS3 (green) (b); dsRNA and NS5 (green) (c);(B) dsRNA and Cav-1 (green) (a); or dsRNA and flotillin (green) (b). Colocalization of dsRNA was observed at different times after infection. (C)HMEC-1cells were infected with DENV-2 and double stained at different times after infection (18 h and 24 h) dsRNA, red, Cav-1, green; and mock infected, not shown. Nuclear DNA was counterstained with DAPI, and the merged image is shown at higher magnification.

Upon infection, the dengue virus alters cellular membrane structures. Thus, it is possible that the replication complex of the dengue virus is recruited to lipid raft structures, which then provide a platform for RNA replication in cellular membranes, as has been observed for the hepatitis C virus [24]. To determine whether the DENV replication complex localized in lipid rafts, we analyzed the colocalization of Flt-1 and Cav-1 with viral dsRNA. We first investigated whether NS3, NS5, and NS2B colocalized with dsRNA because the first two proteins are part of the replication complex (Fig. 5A a–c). HMEC-1cells were infected for up to 48 h (after the first replication round), fixed, and analyzed by immunofluorescence. Interestingly, NS2B showed a high level of colocalization with dsRNA, even though NS2B was previously shown not to be a part of the replication complex [25]. In contrast, NS3 showed equal levels of colocalization with dsRNA, although no clear overlap was observed for the NS5 protein anddsRNAin the cytoplasm. Next, cells were double stained to evaluate the colocalization of dsRNA with Cav-1 and Flt-1. In contrast to the colocalization observed between viral proteins and lipid raft markers, a small amount of overlap between dsRNA and Cav-1 or Flt-1 was observed by immunofluorescence, in both cases with a clustered distribution (Fig. 5B a–b).

To eliminate the possibility that localization varies depending on the time after infection, the kinetics of infection was assessed, and colocalization assays were conducted to determine the locations of DENV dsRNA and Cav-1. Colocalization of Cav-1 and dsRNA was weak throughout the time course (Fig. 5C 18–24 h post infection). These results suggest that dsRNA staining may represent the RNA replication complex (Fig. 5). The colocalization of TfR with viral dsRNA and NS3 was also analyzed, but colocalization was not observed (data not shown). Considering that NS3, NS2B, and NS5 are involved in the viral replication complex, the above observations suggest that parts of replication and DENV polyprotein processing occur in lipid rafts.

### Association of Cav-1 and NS3 with lipid rafts

**Figure 6:**
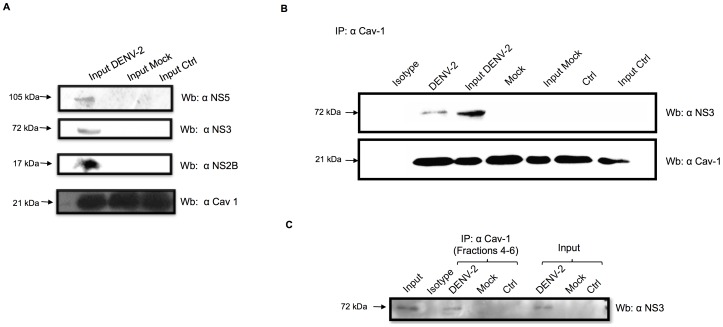
Association of NS3 with caveolin. (A) HMEC-1cells were infected with DENV-2 at 10 MOI for 48 h. The cells were lysed, and lysates were divided into two aliquots. One aliquot was used for western blotting with anti-NS2B, anti-NS3, anti-NS5, and anti-Cav-1 antibodies. (B) The other aliquot was used for immunoprecipitation with anti-Cav-1 and developed with anti-NS3 antibody.(C) a experiment performed as described above the lysed was subjected to sucrose gradient ultracentrifugation The floating bands corresponding to lipid rafts (3, 4, and 5) were mixed and used in immunoprecipitation assays with an antibody targeting α-Cav-1. Immunoprecipitated samples were analyzed by western blotting with rat-anti NS2B, rat monoclonal anti-NS5, and mouse monoclonal anti-NS3 and anti-Cav-1 antibodies.

Considering that NS3 and NS2B colocalized with Cav-1 over the 36-h infection and that they were consistently present in DRMs, we hypothesized that the processing of DENV proteins is associated with caveolar lipid rafts. First we infected HMEC-1 cells and at 48 hour were lysed and analyzed by western blot with anti-NS2B, anti-NS3, and anti-NS5 antibodies(Fig. 6A)To analyze the direct interactions between the viral proteins NS2B, NS3, and NS5 with Cav-1 or Flt-1, the infected cells were lysed and immunoprecipitated with Cav-1 and then analysed by western blot with anti NS2B, anti NS3 and anti NS5, but we only observed a band of 72 kDa (Fig 6B). This band was enriched in lysates from infected cells and was absent in lysates from mock-infected and uninfected cells. Furthermore, HMEC-1 cells were infected, and DRMs were isolated by sucrose gradient centrifugation. Then fractions 3 and 4 (in which Cav-1 and Flt-1, are present) were mixed. Again proteins were immunoprecipitated with antibodies specific to Cav-1 and assessed by blotting with anti-NS2B, anti NS3 and anti NS5 antibodies. In Fig. 6Conly a band of approximately 72 kDa corresponding to the NS3 protein was observed. However, when the same immunoprecipitated fraction was analyzed using specific anti-NS2B and anti-NS5 antibodies, no signals were observed. Although NS3 and NS2B colocalized with Flt-1 in confocal analyses, when the anti-Flt-1 antibody was used for immunoprecipitation, we were not able to detect NS2B, NS3, or NS5 with specific antibodies (data not shown). These results suggest that, in contrast to the clear association between Cav-1 and NS3, interaction of Flt-1 with DENV proteins may be weak or non-existent.

### Dependence of late-stage DENV infection on lipid rafts

The data obtained in the present study demonstrated that DENV proteins colocalized with lipid raft-resident proteins. Cholesterol is an important component of lipid rafts. Moreover, previous research has demonstrated the dependence of DENV entrance on cholesterol and a likely role for cholesterol in viral replication[26]. Therefore we investigated the effects of cholesterol depletion on the association of NS3 with lipid rafts by treating cells with methyl-beta cyclodextrin (MβCD), which affects cholesterol distribution and destroys caveolar structures [27]. First, we treated HMEC-1 cells with different concentrations of MβCD (5, 10, 15, or 20 mM) and then measured cytotoxicity using trypan blue exclusion assays to establish the nontoxic concentration. The highest concentration showed slight toxicity during a 48-h treatment (data not shown).

**Figure 7:**
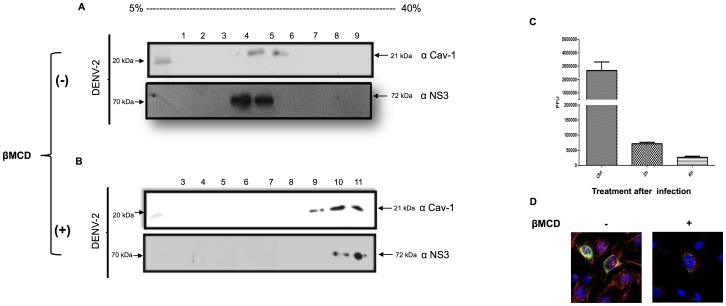
Depletion of cholesterol by MβCD affects DENV-2 infection in HMEC-1. HMEC-1cells were infected at 10 MOI. At 120 min after adsorption, the inoculated virus was washed off, and the cells were cultured in fresh medium. At 2 or 6 h post infection, the cells were mock treated (A) or treated with 10 mM MβCD (B) for 2 h. The drug was then washed off, and the cells were cultured in fresh serum-free medium for 48 h. Detergent-resistant membrane fractions obtained from sucrose gradient ultracentrifugation were collected, and Cav-1 and NS3 were analyzed. The recovered fractions were numbered from 1 (top fraction) to 11 (bottom fraction). (C) The culture supernatants were harvested to determine the viral titration by plaque-forming assays. (D) Disaggregation of lipid rafts after10 mM MβCD treatment.

Using 10 mM MβCD, we evaluated the antiviral effects of MβCD on DENV-infected cells. Polyprotein translation and RNA replication occur in the first 6 h. Therefore, to ensure that lipid raft disruption did not affect the viral entry process, but only subsequent steps in the replication cycle, MβCD was added 2 h and 6 h after removal of the viral inoculums. After 24 hours of incubation, membrane and cytosolic fractions were separated in a sucrose gradient, and the fractions were analyzed. In infected cells, Cav-1 and NS3 proteins were detected mainly in fractions 3, 4, and 5, suggesting that rafts promoted the localization of the molecules to the upper fractions (Fig. 7A). Cholesterol depletion disrupted the lipid rafts, resulting in the diffusion of lipid raft proteins. Both Cav-1 and NS3 were displaced to fractions 9, 10, and 11 in the sucrose gradient (Fig. 7B). Furthermore, the amount of viral protein decreased, indicating a direct effect of cholesterol on viral replication. We also harvested supernatants from the above experiments and performed plaque assays (Fig. 7C). The viral load decreased upon treatment of the cells with MβCD. Cholesterol depletion in infected cells showed a significant reduction in the release of infective DENV particles.

## Discussion

Several studies using different positive-strand RNA viruses have demonstrated that reorganization of the intracellular membrane creates a scaffold for the viral replication machinery [28], [29]. Moreover, during the dengue viral cycle, cell membranes are critical for the entrance, translation, replication, and assembly of viruses. Electron microscopy analysis has shown that DENV-infected cells develop important changes in membrane organization; the changes may be derived from the ER. Furthermore, electron tomography analysis has clearly demonstrated that viral replication takes place on double-membrane vesicles adjacent to the ER [8].

Cell membranes contain lipid rafts, which may provide a specialized microenvironment that enables viruses to recruit or locate cellular molecules, resulting in a successful replication cycle. Previous studies on dengue viruses have demonstrated the importance of the membrane and its lipid raft composition for receptor-mediated or -facilitated early viral entrance [16], [30], [31]. However, the role of lipid rafts in the late phases of the DENV life cycle, including polyprotein processing, viral replication, assembly, and budding, have not been sufficiently evaluated [16].

In HMEC-1 cells infected with DENV-2, NS3 and NS2B colocalized with Cav-1 and, to a lesser extent, with Flt-1, both of which are resident proteins of lipid rafts. This suggests that the events immediately after DENV entrance may be confined to lipid rafts, as has been demonstrated for Japanese encephalitis virus [16]. To evaluate whether processing occurs in lipid rafts, HMEC-1 cells were transfected with the N-terminal sequence of NS3 (NS3pro), which contains the NS3 protease domain [32]. NS3pro colocalized with Cav-1. A recent study performed with a West Nile virus recombinant protein showed that NS3 contains a membrane-binding site [33], [34]. NS2B and NS3 form a protease complex that is necessary for polyprotein processing. The fact that NS2B strongly colocalized with Cav-1 corroborates the hypothesis that Cav-1 restricts the orientation of the protease on the ER membrane, because NS2B is not part of the replication complex [35]. On other hand, Flt-1 is a marker for lipid raft microdomains that are distinct from caveolar lipid rafts. Lipid raft domains containing Flt-1 serve as physical platforms for various molecules that are crucial for intracellular signaling. In the present study, Flt-1 did not directly interact with NS3, NS2B, or NS5 in our HMEC-1cells experimental model.

Additionally, we demonstrated that disruption of lipid rafts by cholesterol depletion with MβCD treatment reduced the release of DENV. MβCDwas administered after the viral RNA was in the cytoplasm. Thus, lipid raft disruption affected the replication and polyprotein processing stages. As a result, viral yield decreased. Consistent with lipid raft disruption, the partitioning of Cav-1 and NS3 in sucrose gradient fractions changed.

The constant but weak colocalization of dsRNA with lipid raft proteins such as caveolin and the lack of colocalization of NS5 (RNA polymerase) with Cav-1 and Flt-1suggest that replication does not occur in lipid rafts. However, co-immunoprecipitation and pull-down assays with recombinant proteins in JEV- [36] and DENV-infected [37] cell lysates have demonstrated that NS3 and NS5 interact. Therefore, replication might occur in membranes, and molecules such as NS3 and NS4A might be anchored, while the polymerase might not be anchored. Furthermore, protein-protein interactions might facilitate the transport of non–raft-associated proteins to lipid raft microdomains. Therefore, NS5 might not necessarily interact with lipid rafts during viral replication, as has been described for the influenza virus M1 protein, a non–raft-associated protein, which interacts with hemagglutinin and neuraminidase [38].

In the current study, we demonstrated a direct interaction between NS3 and Cav-1 using immunoprecipitation assays. NS3 recruits fatty acid synthase; the direct interaction between these proteins is associated with increased fatty acid biosynthesis in DENV-infected cells. Additionally, *de novo*-synthesized lipids preferentially co-fractionate with DENV RNA. The interaction between DENV RNA and fatty acid synthase helps establish the DENV replication complex. [39].

The role of Cav-1 during DENV polyprotein processing has not been elucidated. However, in other viruses, Cav-1 serves as a scaffolding protein that organizes and sequesters signaling molecules within caveolar membrane-specific lipids (e.g., cholesterol and glycosphingolipids). Cav-1 regulates caveolae-dependent functions, and interaction of cytoplasmic proteins with Cav-1 is sufficient to target these proteins to caveolar raft microdomains [40] and in lipid droplet structures, where replication has been described. Furthermore, lipid rafts serve as sites of assembly and budding for other enveloped viruses, such as influenza and HIV [28].

The data presented above strongly suggest that lipid rafts are involved in viral processing and replication. The interaction between Cav-1 and DENV NS3 during processing and replication appears to constitute a signaling or regulatory event that recruits other cellular molecules required for processing or replication. Recent data have demonstrated that interaction of Cav-1 with hemagglutinin and M proteins from the parainfluenza virus (paramyxovirus family) enhances the clustering of viral complexes at the plasma membrane and triggers viral budding from caveolae [41]. From this perspective, the presence of caveolin in the DENV membrane remains to be clarified. Cav-1 is considered an important signaling molecule and is the focus of many studies. Recent studies have demonstrated that Cav-1 blocks HIV replication through nuclear factor kappa-light-chain-enhancer of activated B cells [20].

Caveolar rafts appear to be suitable locations for various processes in the viral life cycle. Our results indicate that viral proteins involved in polyprotein processing may be located in lipid rafts. Therefore, interactions with the membrane may affect the activity and substrate selectivity of NS3 protease. By demonstrating that the interaction between NS3 and the membrane during polyprotein processing may involve the scaffolding protein Cav-1, our results corroborate a recent study that identified NS3-membrane interactions [34].To the best of our knowledge, this is the first study to address the implications of caveolar lipid rafts in DENV infection after virus entry.

## Materials and Methods

### Cell culture and DENV-2 virus

HMEC-1 (HMEC line 1; Centers for Disease Control, Atlanta, GA, USA) were grown at 37°C under 5% CO_2_ in MCDB131 medium (Gibco/Life Technologies, Carlsbad, CA, USA) supplemented with 10% fetal bovine serum, 1 µg/mL hydrocortisone (Sigma Aldrich, St. Louis, MO, USA), 10 ng/mL epidermal growth factor (Gibco), 100 U penicillin, and 100 mg/mL streptomycin. Cells were detached by treatment with 1000 U/mL trypsin and 0.5 mM EDTA.

The DENV-2 clinical isolate and its stock preparation and titration have been described previously [42]. The virus was titrated by the standard plaque-forming assay technique using BHK-21 cells as described previously. After 5 days, the resulting plaques were stained with naphthol blue-black solution to quantify the plaque-forming units [43].

### Antibodies

The mouse monoclonal antibody directed against NS3 (D-7) and the rat monoclonal antibody against NS5 (13G7) have been previously described [44], [45].Rabbit polyclonal anti-Cav-1 and mouse monoclonal anti-Flt-2 antibodies were obtained from Santa Cruz Biotechnology (Santa Cruz, CA, USA). Rat polyclonal antibodies targeting NS2B and NS3 were produced in our laboratory. Mouse monoclonal anti-TfR antibodies were obtained from Zymed (San Francisco, CA, USA). Mouse monoclonal anti-dsRNA antibodies were obtained from English & Scientific Consulting (The data obtained in the present study demonstrated that DENV proteins colocalized with lipid raft-resident proteins. Cholesterol is an important component of lipid rafts. Moreover, previous research has demonstrated the dependence of DENV entrance on cholesterol and a likely role for cholesterol in viral replication [26]. Fluorescein isothiocyanate goat (1∶100) and Texas Red-labeled goat anti-mouse antibodies were also used.

### Infection of HMEC-1 cells and immunofluorescence analysis

HMEC-1 cells were trypsinized and resuspended in MCDB131 medium. Cells were then seeded on glass coverslips (1×10^5^ cells/mL). After 24 h, the culture medium was removed and the monolayer was washed. Active or UV-inactivated DENV-2 was then added at 10 MOI. The cells were incubated at 37°C for 120 min, and the inoculum was then removed. The cells were washed, and fresh growth medium was added. Infected cells were analyzed by immunofluorescence or western blotting at different times after infection, as described previously [32]. Briefly, the cells were fixed with 4% paraformaldehyde (Sigma-Aldrich) in phosphate-buffered saline (PBS) for 20 min at room temperature. The cells were then permeabilized with 0.1% Triton-X 100 in PBS and blocked with 10% normal goat serum. The cell monolayer was treated for 60 min with primary mouse monoclonal anti-NS3 [44], rat polyclonal anti-NS3, rat polyclonal anti-NS2B, polyclonal rat anti-NS5, mouse monoclonal anti-Flt-2, rabbit polyclonal anti-Cav-1, or mouse monoclonal anti-dsRNA (monoclonal antibody J2) antibodies, followed by treatment with fluorochrome-conjugated secondary goat anti-mouse IgG1 (1.5 µg/mL), PE-conjugated goat anti-rabbit IgG (1 µg/mL), or Texas Red-conjugated goat anti-rat IgG (1 µg/mL). An irrelevant isotype antibody that matched the monoclonal antibody was used as a negative control. Finally, nuclei were labeled with DAPI (1 µg/mL) in PBS for 10 min, and the slides were mounted with Vectashield (Vector Laboratories, Burlingame, CA, USA). Images were captured using two different confocal microscopes (Leica SP2 and OLYMPUS FVX). The slides were analyzed with a Leica SP5 confocal microscope. Images were captured using a 63×1.3 NA oil-immersion objective.

### Extraction of lipid rafts by Brij sucrose gradient fractionation

The membrane was separated into Triton-soluble and -insoluble components as previously described [16]. Briefly, 48 h after infection, the cell monolayer was scraped in ice-cold PBS and centrifuged at 2065×*g*. The pellet was immersed in 1 mL TNEV buffer (150 mMNaCl, 25 mMTris-HCl, pH 7.5, 5 mM EDTA, 1% Brij, and a cocktail of protease and phosphatase inhibitors (Roche, Mannheim, Germany) and stored at 4°C for 40 min on ice. The lysates were homogenized on ice with 20 strokes in a pre-chilled Dounce homogenizer. After centrifugation at 3442×*g* for 11 min at 4°C, the supernatant was applied to a discontinuous sucrose gradient. The supernatant was mixed with 1 mL TNEV-85% sucrose and 6 mL TNEV-45% sucrose and was then overlaid with 3.5 mL TNEV-5% sucrose. The gradient was centrifuged at 55,000×*g* for 20 h at 4°C in a SW40Ti rotor (Beckman, Pasadena California, USA). Eleven 1-mL fractions were collected from top to bottom of the gradient and diluted in loading buffer. Next, 40 µL samples were subjected to sodium dodecyl sulfate-polyacrylamide gel electrophoresis (SDS-PAGE) and western blot.

### SDS-PAGE and immunoblotting

Each 50-µL fraction was mixed with 10 µL of Laemmli loading buffer and then heated at 95°C for 10 min. Sample proteins were resolved by SDS-PAGE using 12% Tris-HCl gels for 80 min at 160 V (Mini-Protean Cell; Amersham Biosciences, Piscataway, NJ, USA) and then electrotransferred (120 V for 2 h) onto nitrocellulose membranes (Hybond ECL; GE Healthcare, Little Chalfont, UK). Air-dried membranes were blocked and then incubated with the appropriate primary antibody, followed by the appropriate horseradish peroxidase (HRP)-conjugated secondary antibody (1∶3000) in PBS plus Tween-20. The membranes were stripped if necessary. After further washing with PBS plus Tween-20, the membranes were treated with Western Lightning Enhanced Chemiluminescence Reagent (Pearce, Rockford, IL, USA), and immunoreactive proteins were detected by exposure to film (Kodak, Rochester, NY, USA).

### Immunoprecipitation

For immunoprecipitation, 500 µL of lysates of HMEC-1 cells were pre-cleared with 50 µL protein G-Sepharose beads (Invitrogen, Carlsbad, CA, USA) for 16 h at 4°C. The beads were then centrifuged, and the supernatant was recovered and mixed with 2 µg of rabbit monoclonal anti-Cav-1 antibody (Santa Cruz Biotechnology, Dallas Texas, USA). This mix was incubated for 12 h at 4°C with rotation and immobilized on protein G-Sepharose beads for 16 h at 4°C. The beads were then washed four times with lysis buffer containing protease inhibitors. Finally, the complex was boiled in Laemmli buffer containing 5% β-mercaptoethanol (β-ME; Sigma, St. Louis Missouri, USA). Immunoprecipitated proteins (25 µL) were loaded on 15% polyacrylamide gels, electrophoresed, and transferred to nitrocellulose (Bio-Rad, Hercules California USA). The membranes were blocked with 5% skim milk and incubated with mouse monoclonal anti-NS3 [44]antibody overnight at 4°C, followed by incubation with anti-mouse-HRP-conjugated antibodies. Protein bands were detected using chemiluminescence. For total lysate analysis, 15 µg of protein was loaded per lane, and western blot analysis was performed as described above.

### MβCD treatment of DENV-infected HMEC-1 cells

HMEC-1cellsgrown in a monolayer were washed and then infected with 10 MOI DENV for 2 h. After removal of the viral inoculum, the cells were washed and incubated at 37°C. Two or six hours later, the cells were washed exhaustively with serum-free medium and were either mock-treated or treated with 10 mM of beta-methyl ciclodextrine (MβCD Sigma-Aldrich) for 2 h at 37°C in serum-free medium. The concentration of MβCD was determined from a dose-response curve. After two washes with serum-free medium, the cells were grown in serum-free medium alone. After 48 h of incubation, cell culture supernatants were harvested. The number of infectious viruses was quantified by the plaque-forming assay, and cells were lysed to analyze protein concentrations for western blotting.

### Transfection

The sequences encoding NS3pro and NS2BNS3 were fused to GFP to create the plasmids pEGFP/NS3pro and pEGFP/NS2BNS3pro as described previously [32]. HMEC-1 cells (2×10^5^) were plated 24 h before transfection. Briefly, HMEC-1 cells were grown to 50% confluence in 6-well plates, the medium was removed, and the cells were transfected with pEGFP/NS3pro, pEGFP/NS2BNS3pro, or the parental vector pEGFP-N1 using 1 µg of DNA and 1 µL of Lipofectamine 2000 reagent (Invitrogen Life Technologies) mixed in 100 µL of serum-free Opti-MEM for 4.5 h at 37°C. The cells were then cultured in complete medium, and the expression of NS3pro-GFP, NS2BNS3pro-GFP, and pEGFP-N1 was verified by detection of EGFP with fluorescence microscopy 24 h after transfection.
